# Rotationplasty for Spindle Cell Tumor of Tibia

**DOI:** 10.7759/cureus.20850

**Published:** 2021-12-31

**Authors:** Husnain Khan, Nur Ul Ain, Hashaam Khurshid, Dujanah S Bhatti

**Affiliations:** 1 Plastic Surgery, Holy Family Hospital, Rawalpindi, PAK; 2 Plastic and Reconstructive Surgery, Holy Family Hospital, Rawalpindi, PAK; 3 Plastic and Reconstructive Surgery, Rawalpindi Medical University, Rawalpindi, PAK; 4 Surgery, Aberdeen Royal Infirmary, Aberdeen, GBR

**Keywords:** cancer, plastics surgery, tibia, spindle cell lesion, rotationplasty

## Abstract

Rotationplasty has gained popularity for lower limb salvage in oncological resection in place of amputation. It provides more reliable and functional results, with overwhelming cosmetic concerns. We discuss the use of this functional and oncologically reliable technique for an 18-year-old male patient who presented with malignant spindle cell carcinoma. With a multidisciplinary team (MDT) approach and involvement of occupational therapy and rehabilitation, we achieved satisfactory results with no discernible impact on the social and emotional functioning of our patient.

## Introduction

Limb salvage has gained popularity over amputation for malignant tumors of lower extremity among oncological surgeons to gain tumor-free margins and to maintain limb function. Rotationplasty has been widely used as an alternative to above-the-knee amputation and has a satisfactory outcome.

Rotationplasty is to shorten the leg and rotate it by 180 degrees, allowing the ankle to function in place of a knee joint. It is a dependable and durable alternative to above-the-knee amputation for the treatment of malignant tumors in the knee. Patients who have undergone rotationplasty are more active and they participate in higher demanding activities, as compared with patients who have had an above-the-knee amputation. Rotationplasty is infrequently performed in adults despite the low complications rate associated with this procedure.

In this study, we discuss rotationplasty for the treatment of malignant spindle cell tumor of the upper right tibia, involving the knee joint.

## Case presentation

**Figure 1:**
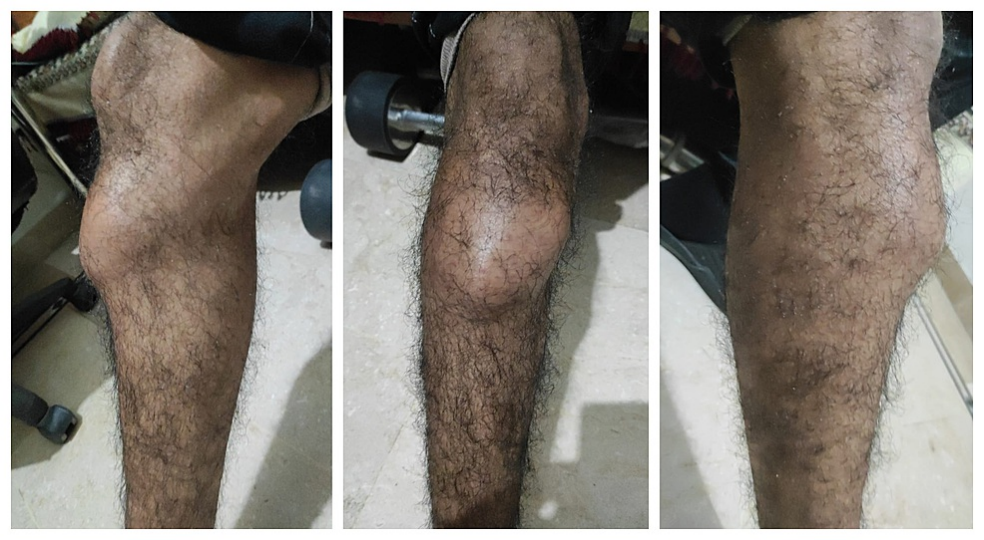
Pictures of the involved knee. Three view pictorial representation of the involved right knee. Left: Medial view Middle: Anterior view Right: Lateral view

A young male, 18 years of age presented to us with a malignant spindle cell tumor of the right tibia involving the knee joint in February 2021. He had a history of swelling around the right knee joint for the last six months (Figure 1). Previous work up and MRI was consistent with biopsy-proven spindle cell tumor of the tibia. It had involved the proximal head of the tibia and was 3 mm away from the knee joint cavity.

Different therapeutic options were given to the patient, including mega-prosthesis, resection of joint and knee arthrodesis, Van ness rotationplasty, and above-the-knee amputation. The patient was made aware of all the available treatment modalities with detailed informed consent.

**Figure 2:**
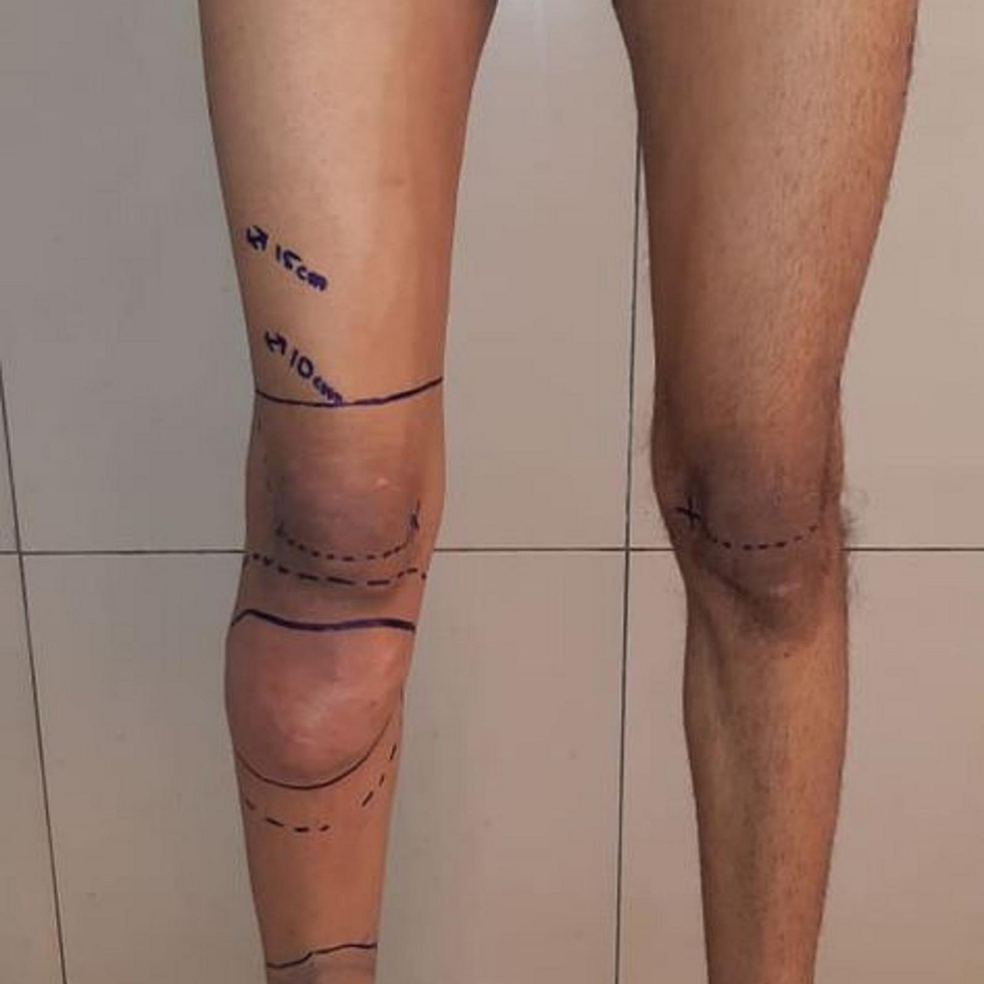
Pre-operative marking of the right knee for tumor resection and knee rotationplasty, the amount of femur to be taken out should correspond to the amount of tibia that is preserved.

The patient was marked preoperatively as shown in Figure 2. 

The patient decided to opt for rotationplasty after discussing the financial restraints and functional outcome of each of the procedures. The patient was shown videos and pictures of different individuals who had gone through the same procedure. Preoperative workup was done with the planning of the oncological resection and involvement of sciatic nerve was excluded. Multidisciplinary team (MDT) was involved throughout the case. Rotationplasty (Van ness procedure) was performed in February 2021 and en bloc resection of the distal femur and proximal tibia with knee joint was done, along with intra-medullary nail fixation by the orthopedic team. The drain was placed which was removed on the third day, and recovery was uneventful.

**Figure 3:**
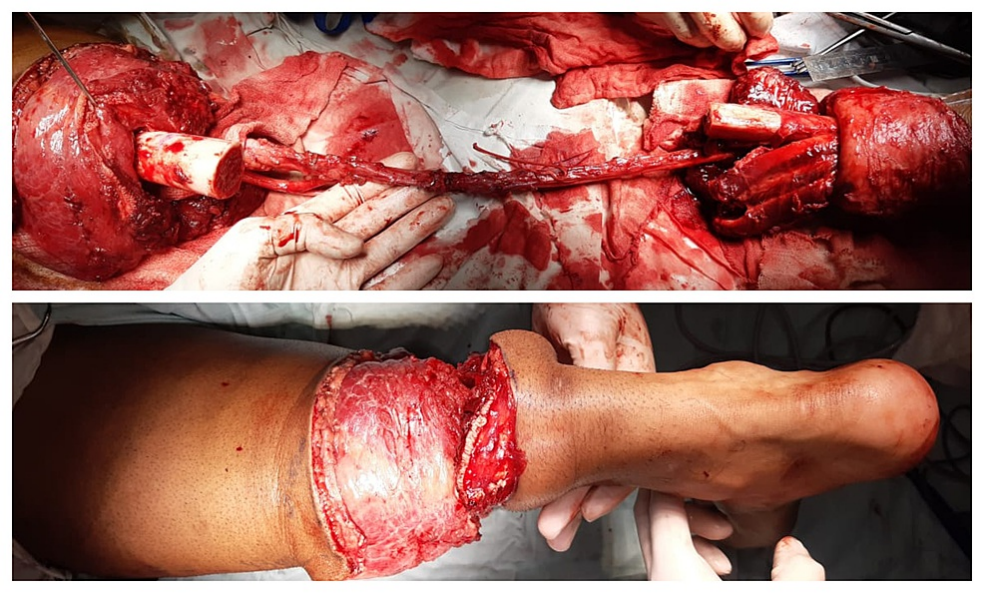
Per-operative picture. Above: After excision of giant cell tumor while maintaining the neurovascular bundle to keep the foot viable Below: After knee rotationplasty, foot has now been reversed and ankle joint now plays the role of pseudo knee joint

**Figure 4:**
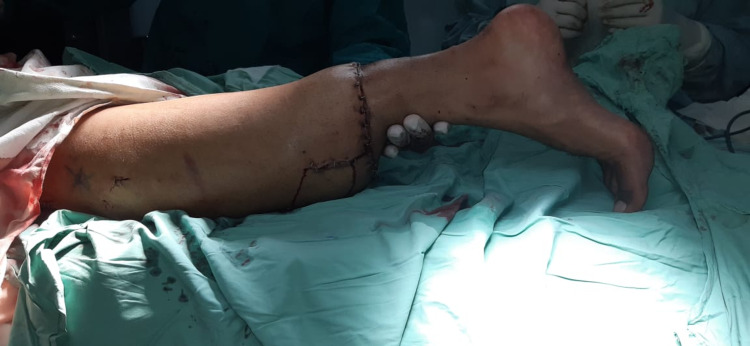
Post op result after skeletal fixation and skin closure.

After excising the tumor, the neurovascular bundle was preserved (Figure 3) and his foot was revered. The ankle joint replaced the knee joint (Figure 4). 

Soft tissue healing occurred within one month. The range of motion improved with physiotherapy within two months. The patient's activity was partial weight-bearing after two months and full weight-bearing with prosthesis after four months.

At last follow up, five months after the surgery the patient has had three cycles of chemotherapy with three cycles remaining. His radiograph shows adequate healing of femoro-tibial bony union. His prosthesis was planned for next month, and the patient was satisfied with his functional outcome. He has come to terms with the cosmetic appearance of the limb. He can walk 50 m with crutches without pain, and he can climb stairs with crutches, and he has started partial weight bearing on his affected limb.

## Discussion

Rotationplasty was first described by Borggreve for limb shortening and knee joint ankylosis secondary to tuberculosis in 1930 and then later on Van Ness popularized this procedure for proximal femoral focal deficient [1-2]. In 1982, Kotz and Salzer described promising outcomes in patients of osteosarcoma in the distal femur who underwent tumor resection and rotationplasty [3]. It also played a role in the treatment of congenital femoral deficiency [4]. Today, rotationplasty can be recommended in patients diagnosed with extensive soft tissue tumors, failed limb-salvage procedure, or as an alternative to endoprosthesis [5-6]. In skeletally immature patients with a malignant tumor around the knee, rotationplasty offers a reliable choice for tumor resection [7]. The current recommendation for tumor cases is favored towards rotationplasty, offering a wide surgical margin resection with a functional remaining limb [8].

Rotationplasty is a limb-sparing procedure that preserves the foot and consists of en bloc resection of the knee joint, distal femur, and proximal tibia while retaining the femoral artery and sciatic nerve and rotation of the distal segment 180 degrees so that the reversed ankle functions like a knee joint and the foot functions as the tibia that can be fitted with a below-knee type prosthesis [9-11]. This new knee has active flexion of nearly 90 degrees and has a short rehabilitation period with a prosthesis [6]. Winkelmann and colleagues classified rotationplasty into five groups and noted that the procedure can be used not only in children with a sarcoma of the distal femur but also to treat tumors in the proximal femur and proximal tibia [12].

Patients with rotationplasty limbs have exceptional psychological and functional outcomes, including physical and mental functioning, vitality, somatic pain, emotional, and social health [8, 13-16]. Rotationplasty is a good alternative for above-the-knee amputation [17]. It avoids the complications associated with amputation stumps, for instance, phantom pain or neuroma. The reconstructed limb has satisfactory weight-bearing capacity when the prosthesis is worn [17]. The functional advantage of rotationplasty is a more efficient gait with the ability to run, climb stairs, etc., and lower oxygen consumption than above-the-knee amputation or knee arthrodesis [18-19]. The main problems with rotationplasty are cosmetic appearance and potential psychologic issues [6, 8]. However, compared to amputations these patients do not consider themselves as an amputee, and with good functional outcomes, they fare better than the patients who have had an amputation [8].

Rotationplasty is a valid, efficacious, and substitute procedure to above-the-knee amputation or endoprostheses, especially for young patients, and allows patients to recuperate near-normal functional performance and patient satisfaction.

## Conclusions

Our aim in this patient was to find the best treatment option for him so that he can regain near-normal function after resection of the tumor around his knee joint. In rotationplasty, the ankle adopts the role of a functioning knee joint as it is rotated 180 degrees, enabling the patient to start the rehabilitation protocol. One of the benefits of Van Ness rotationplasty is the lack of phantom pain because of sciatic nerve preservation. Moreover, compared to above-the-knee amputation there is less incidence of infection, less restriction on daily life activities, better gait, and better proprioception in rotationplasty. Although it is a complex procedure, its advantages make it a desirable treatment option. We have concluded from our experience that rotationplasty is a valid, efficacious, and a substitute procedure to above-the-knee amputation or endoprostheses, especially for young patients, and allows patients to recuperate near-normal functional performance and patient satisfaction.
